# Experimental investigation on partial cement replacement with binary blended bagasse ash and calcined dolomite for enhanced C-25 grade concrete performance

**DOI:** 10.1038/s41598-025-98019-7

**Published:** 2025-07-02

**Authors:** Dereje Tesfaye Woldesenbet, Jemal Jibril Mohammed, Solomon Demiss Negedu, Jose Henriques, Endrias Adane Bekele

**Affiliations:** 1 Faculty of Materials Science and Engineering, Jimma Institute of Technology, Jimma University, Jimma, Ethiopia; 2 Faculty of Civil and Environmental Engineering, Jimma Institute of Technology, Jimma University, Jimma, Ethiopia; 3 School of Materials Science and Engineering, Bahir Dar Institute of Technology, Bahir Dar University, P.O. Box. 26, Bahir Dar, Ethiopia; 4 CERG, Faculty of Engineering Technology, Hasselt University, Hasselt, Belgium

**Keywords:** Bagasse ash, Pazzolona, Calcined dolomite, C-25 grade concrete, Compressive strength, Partial replacement, Materials science, Nanoscience and technology

## Abstract

Cement is a globally produced building material and a crucial component of every construction project. Alternative materials, mostly agro-industrial wastes, are emerging as potential cement replacements due to high carbon dioxide emissions associated with cement manufacturing and rising cost of cement. Numerous naturally occurring materials like rice husk ash, corn cob ash, fly ash, slag, silica fume, bagasse ash (BA), and natural pozzolans are used as partial substituents for cement in concrete and mortars due to their strength, cost-effectiveness, and environmental benefits. BA is frequently used as a partial cement replacement in concrete, but most studies limit its utilization to 15%, highlighting the limitations of pozzolanic materials. This study investigates the effects of binary blended BA and calcined dolomite powder (CDP) as partial cement replacement, i.e., 0%, 5%, 10%, 15%, 20%, 25%, 30%, 35%, 40%, 45%, and 50%, on the compressive strength of C-25 grade concrete. Additionally, the physicochemical properties of BA, CDP, and the binary mixture were studied using X-ray diffraction (XRD), Fourier transform infrared spectroscopy (FTIR), scanning electron microscope (SEM), X-ray fluorescence (XRF), and dynamic light scattering (DLS). Furthermore, the effects of the binary mixture on workability, setting time, compressive strength, strength activity index (SAI), water absorption, and dry density on the concrete were evaluated in detail. The compressive strength was examined by casting 66 standard cubes of 15 cm $\:\times\:$ 15 cm $\:\times\:$ 15 cm size and curing them for 7 and 28 days. The compressive strength test indicates that by reducing pozzolana particle size below cement grade and blending BA with CDP, up to 30% of cement can be replaced by enhancing the compressive strength to 36.7 MPa at the end of 28 days.

## Introduction

Concrete, a widely used construction material globally, is experiencing a significant increase in demand due to population growth, urban expansion, and infrastructure development. A significant revolution in the cement and concrete industry is needed for sustainable growth and environmental protection, particularly in reducing carbon dioxide (CO_2_) emissions ^1^. Cement is a chemical compound utilized in construction as a binder, forming bonds between materials through setting, hardening, and binding ^2^. Cement manufacturing contributes significantly to global environmental concerns, emitting 5–10% of CO_2_ and consuming up to 80% of power. Studies explore alternative waste materials to cement for CO_2_ reduction, highlighting the cost-effective and environmentally responsible disposal of waste in the construction industry ^3^. The construction sector faces threats from rising energy production prices, lower CO_2_ discharges, and poor-quality components, necessitating sustainable solutions to reduce environmental impact. It is crucial to explore eco-type cementations materials to replace or partially replace cement to decrease energy consumption and CO_2_ emissions. The need for environmentally friendly energy-building materials has led to extensive research into alternative materials that can mitigate the environmental impact of cement ^4–6^. Researchers have demonstrated the significance of agro-waste ash in achieving high-strength concrete by partially replacing 10–30% of cement with agro-wastes ^2^. Natural materials like limestone powder, marble dust, rice husk ash, corn cob ash, fly ash, slag, silica fume, bagasse ash, and natural pozzolans are partially replacing cement in concrete and mortars ^7^. Raj Bhosale et al. ^8^ studied the strength of rice husk ash and BA as partial cement replacements. They found that using 20% RHA and 20% BA, or 40%, provides the highest compressive strength. Mostafa Shaaban ^5^ study on a binary binder system containing calcined dolomite powder and rice husk ash found that CDP-RHA had superior mechanical properties than OPC, with a mixture containing 50% CDP and 50% RHA being optimal. Muliye Tarekegn et al. ^9^ conducted a study on concrete made from hybrid coffee husk ash and sugarcane bagasse ash, finding that up to 10% of the ash had superior compressive and tensile strength, thereby reducing construction costs and environmental pollution.

### Bagasse ash (BA)

Sugarcane (Saccharum spp. hybrid) is a monocotyledonous crop cultivated in tropical and subtropical regions, is crucial for sugar production, bioenergy, and other derivatives. Its ability to store a high concentration of sucrose in its stem internodes makes it a significant industrial crop ^10^. Brazil leads the world in sugarcane production, with annual crop yields of 814.9 million tons, followed by India at 376.1 million tons and China at 138.3 million tons ^11,12^. In Ethiopia, the sugar industry utilizes only sugarcane and has significantly contributes to the country’s socio-economic development. Although the crop is not native to Ethiopia, it was grown in some parts of the country even before large-scale commercial plantations and the establishment of a modern sugar factory at Wonji-Shoa, Metahara, Finchaa, Tendaho, Arjo-Dedessa, and Kessem primarily for local consumption ^10,13,14^. On average, these companies produce 893,270 tons of sugarcane bagasse annually ^15^. Sugarcane bagasse (SCB), a fibrous by-product of refined sugarcane juice extraction, is utilized as a raw material for paper manufacturing. BA is a potential pozzolanic material that is a byproduct of sugar and alcohol production, is a silica-rich, readily available substance produced by calcining SCB ^16^. However, currently, bagasse is primarily utilized as a fuel propellant in cogeneration boilers for steam production and energy in sugar mills for sugarcane juice processing, typically burned at 500-550 °C for maximum calorific value ^17^. BA demonstrated superior reactivity in pozzolanic assessment tests due to its larger specific surface area, higher amorphous content, smaller particle size dispersion, and lower quartz contamination ^18^. Pozzolanas are naturally occurring materials composed of a very fine dispersion of aluminous and siliceous compounds, in the presence of water that react with Ca (OH)_2_ to form cementation materials ^19–22^. Numerous studies have explored the use of low-cost, agro-industrial waste and green materials to partially replace cement in the construction industry, with BA up to 10% providing the highest compressive strength value ^17–19^. The major reason for this limited percent replacement is primarily due to its chemical composition, which consists mainly of alumina and silica, but minimal calcium oxide ^23^.

### Dolomite powder

Dolomite is a rock-forming mineral composed of calcium and magnesium carbonate, with a theoretical percentage of 45.7 MgCO_3_ and 54.3 CaCO_3_, making it a double carbonate ^24,25^. The pure dolomite powder undergoes calcination to create calcined dolomite, which is then broken down into calcium oxide (CaO) and magnesium oxide (MgO) components. In 2016, Guillaume Jauffret studied the addition of pozzolanic elements to Portland cement using thermally activated dolomite, substituting half-burned dolomite powder for 20-25% of cement and conducting a compressive strength test after 28 days. The highest compressive strength values were achieved at a substitution level of 10-15 mass% half-burned dolomite ^26,27^. However, before blending and partially replacing cement, BA and CDP must be fine and homogeneous to meet industry requirements ^28^. The optimal use of grinding and classifying equipment in mortar and concrete necessitates proper operation and regulation before they can be added to cement for optimal performance ^29^. This work involved grinding BA and CDP using a ball miller, controlling ball diameter and time, to produce ultrafine powder. Grinding increases powder particle surface area and reactivity, as materials are more reactive in their divided state. The pozzolanic reaction is enhanced when BA has an ultrafine particle size distribution ^30–32^. Therefore, this study examines the effects of binary blended BA and CDP on the compressive strength of C-25 grade concrete. Additionally, the physicochemical characteristics of BA, CDP, and its mixture were characterized by using XRD, FTIR, SEM, XRF, and DLS techniques. Furthermore, the effects of the binary mixture on workability, setting time, compressive strength, strength activity index (SAI), water absorption, and dry density on the concrete were examined in detail.

### Novelty

Previous studies have examined the individual effects of BA and CDP as partial cement replacements, highlighting their significant contributions to the field. This research investigates the effects of adding binary blended BA and CDP as a partial substitution of cement on the compressive strength of C-25 grade concrete. Furthermore, this research utilized prolonged grinding to achieve nano-sized particles, enhancing pozzolanic performance. Dynamic light scattering analysis confirmed polydispersed particles, mostly within the nano-range. In addition to that, this study demonstrates the potential to replace cement by up to 30%, reducing cement consumption and carbon dioxide (CO_2_) emissions, contributing to its nobility.

## Materials and methods

### Materials

#### Bagasse’s ash (BA)

In this study, SCB was collected from the Metehara sugar factory located in the Oromia region, about 200 km away from Addis Ababa, Ethiopia. The density of BA was 1.98 gcm^-^^3^.

#### Dolomite

Dolomite powder (DP) was brought from Dan and Friends dolomite manufacturing factory, which was located in Addis Ababa, Ethiopia. The DP from the factory has particles passing through a 25 μm sieve. The density of the row DP used in this research was 2.68 gcm^-^^3^.

#### Coarse aggregate

This study uses locally accessible coarse aggregates with a maximum size of 20 mm and minimum size of 10 mm, following IS 383-1970 for testing. The 20 mm aggregates were filtered using a 20 mm sieve, and after removing dust and debris, they were cleaned and dried. The physical properties of course aggregate was illustrated in Table 1.

**Table 1:** Physical property of course aggregate.

No	Characteristic	Value
1	Type	Crushed
2	Maximum size	20 mm
3	Minimum size	10 mm
3	Specific gravity	2.71
4	Total water absorption	0.48%

#### Fine aggregate

**Fig. 1:**
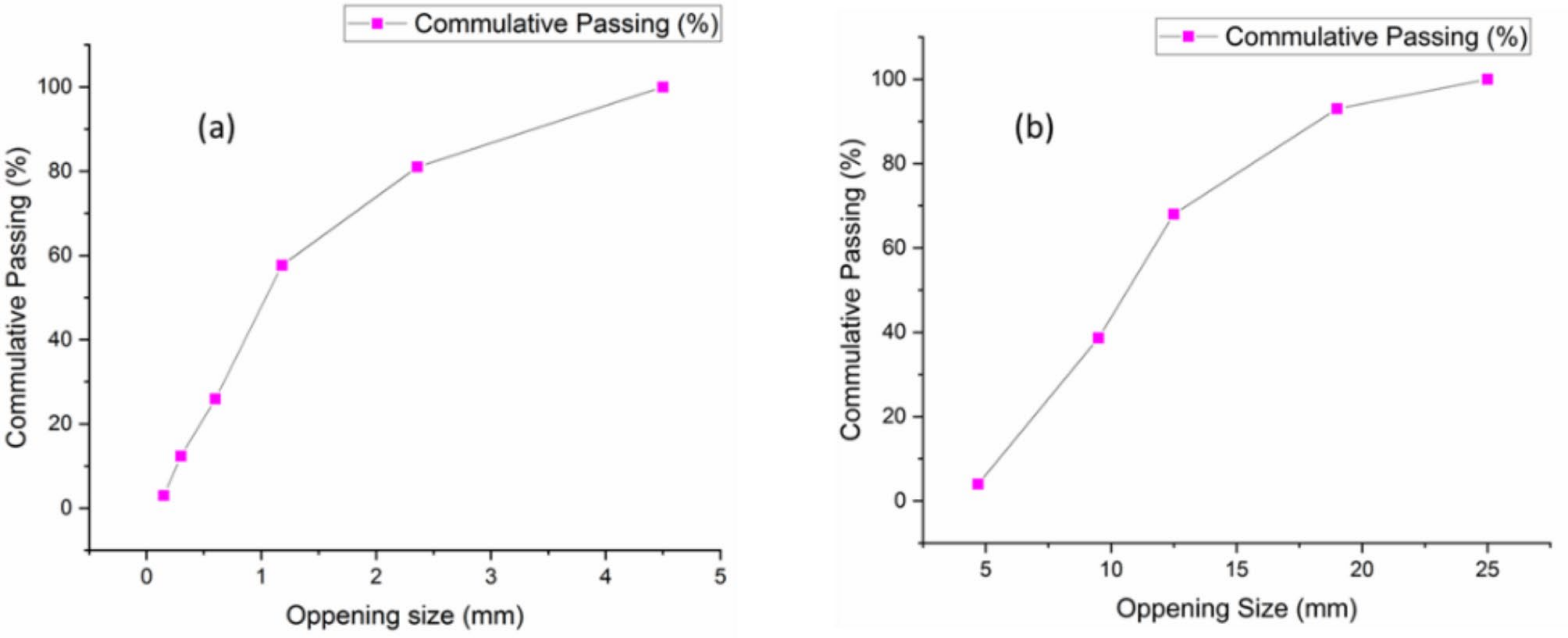
Gradation graph of (**a**) fine and (**b**) coarse aggregates.

Fine aggregates comprise materials like clay, silt, and sand. The experiment utilized locally obtained sand that met IS 383-1970’s Zone-I grading standards. After cleaning, it was filtered through a 4.75 mm screen to remove larger particles, and dried until moisture was lost. Table 2 provides information on the physical properties of fine aggregate. Furthermore, the gradation graph of fine and coarse aggregate was depicted in Fig. 1a, b.

**Table 2:** Physical property of fine aggregate.

No	Characteristic	Values
1	Type	Natural
2	Specific gravity	2.5
3	Water absorption	0.9%
4	Fiennes modules	2.71

#### Cement

The control specimens in this study were made using regular Portland cement (ASTM C150 Type I). OPC Mosobo Grade 43.

#### Water

Concrete cylinders were mixed and cured using water (a drinkable liquid) that was available at the campus laboratory.

### Experimental study

#### Preparation of BA

**Fig. 2:**
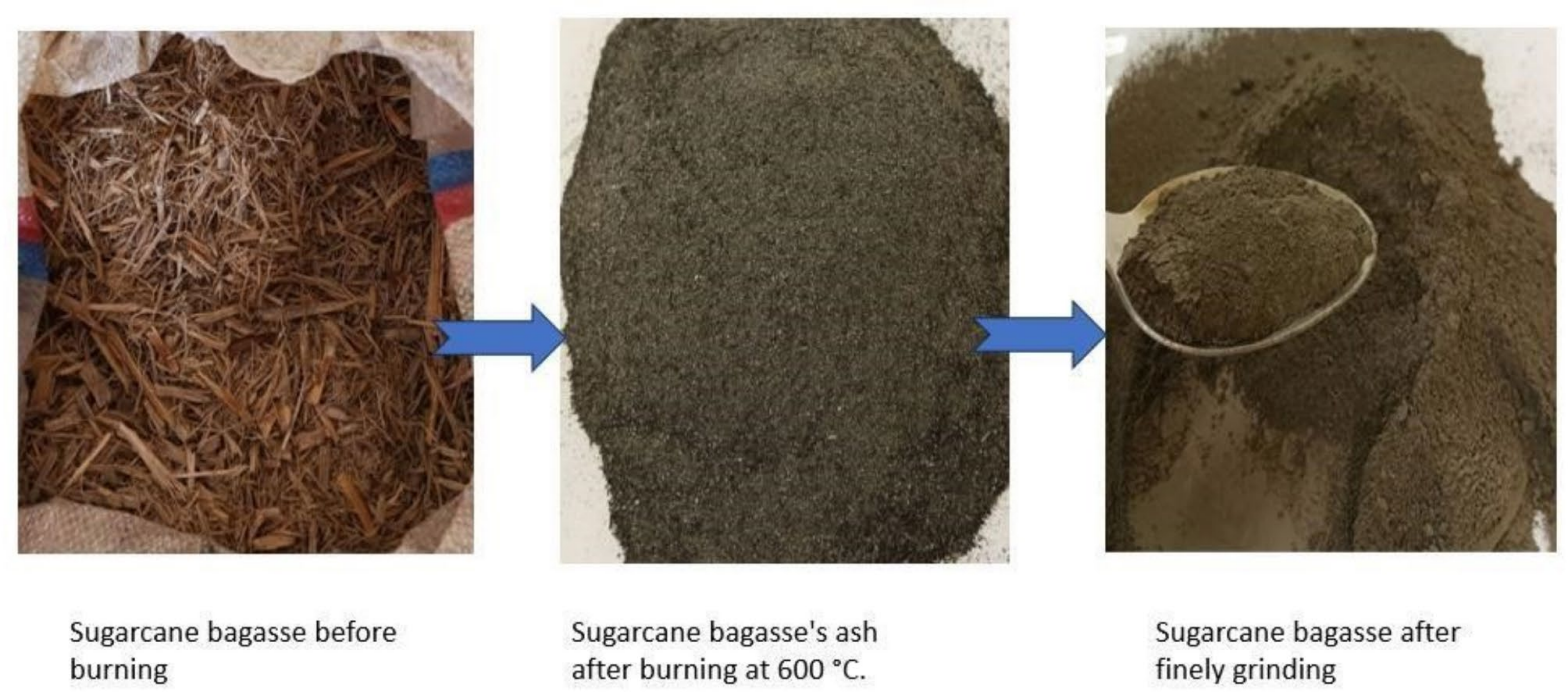
Sample preparation of bagasse ash.

The raw sugarcane bagasse (SCB) collected from the factory was burned in a furnace at 600 °C for 1 h to produce ash, which contains a large amount of amorphous silica with pozzolanic characteristics as shown in Fig. 2. The burned BA was ground into ultrafine particles using a ball miller, and then sieved through 300 μm sieves to separate it from other solid particles. BA, with particle sizes ranging from 0 to 100 μm, was milled using a laboratory planetary ball miller and carbon steel balls with varying diameters with a fixed milling time of 78 min ^33^. The ball size included 15$\:\varnothing\:$, 12$\:\varnothing\:$, 10$\:\varnothing\:$, 5$\:\varnothing\:$, and 0.75$\:\varnothing\:$. The planetary ball miller’s speed was set at 350 rpm.

#### Preparation of CDP

**Fig. 3:**
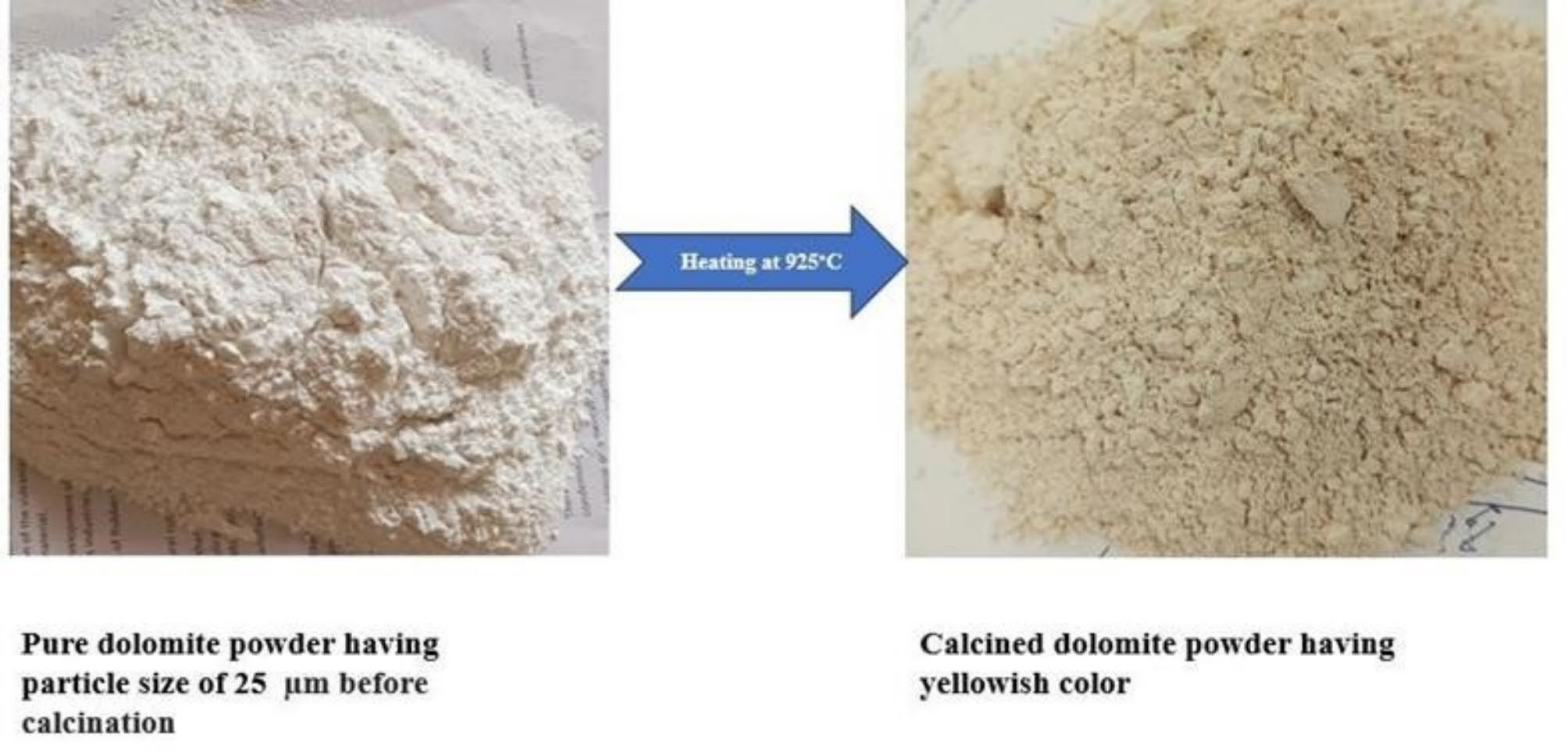
Calcination of dolomite powder at 925 °C.

Dolomite was calcined at 925 °C for 6 h to break down into magnesium oxide (MgO) and calcium oxide (CaO) components (Fig. 3)^25^. After calcination, it was grinded using a planetary ball miller for 39 min to achieve a uniform particle size. The process occurs in a CO_2_ containing atmosphere, with magnesite breaking down at 770 °C and calcite breaking down at 915 °C ^34,35^. The calcination process was shown in Eqs. (1–3):

1 $$\:CaMg{\left({CO}_{3}\right)}_{2}\to\:CaOMgO+{2CO}_{2}$$

2 $$\:{MgCO}_{3}.{CaCO}_{3}+heat\to\:MgO.{CaCO}_{3}+{CO}_{2}$$

3 $$\:{MgO.CaCO}_{3}+heat\to\:MgO\:CaO+{CO}_{2}$$

### Blending of BA and CDP

After carefully preparing, two samples of BA and CDP were blended and grinded in a ball miller for 10 min to form uniformity, with blending performed at 75% BA and 25% CDP by volume.

### Concrete mix design

Concrete properties depend on many factors, such as cement, water, aggregate volume, batching, mixing, placing, compacting, and curing ^36^. This study followed the Indian standard (IS 10262 -1982) in designing the mix for C-25 grade concrete with the aforementioned parameters. A C-25 grade concrete mix was developed without BA and CDP, and BA and CDP were added to replace 5, 10, 15, 20, 25, 30, 35, 40, 45, and 50% of cement, respectively. The total number of cubic samples prepared for the 7th and 28th days compressive strength was 66, with 3 cubes for a single test. The mixing ratio for C-25 grade concrete was 1:1:2 in nominal volumetric mix ratio, with a constant water-cement ratio of 0.5, as shown in Table 3 ^37^.

**Table 3:** Concrete mix design of partial replacement of cement by 5% increment of (SBA/CDP) binary mixtures.

Type	Cement (kg m^− 3^)	SBA/CDP (kg m^− 3^)	Water (kg m^− 3^)	Fine aggregate (kg m^− 3^)	Coarse aggregate (kg m^− 3^)
100% OPC	341	0	171	648	1057
95% OPC/5% (BA/CDP)	323	17	171	614	1001
90% OPC/10% (BA/CDP)	306	34	171	581	949
85% OPC/15% (BA/CDP)	289	51	171	549	896
80%OPC/20% (BA/CDP)	272	68	171	517	843
75%OPC/25% (BA/CDP)	255	85	171	485	791
70% OPC/30% (BA/CDP)	238	102	171	452	738
65% OPC/35% (BA/CDP)	221	119	171	420	685
60%OPC/40% (BA/CDP)	204	136	171	388	632
55%OPC/45% (BA/CDP)	187	153	171	355	580
50%OPC/50% (BA/CDP)	170	170	171	323	527

### Concrete casting

After concrete mix design, different C-25-grade 43 concerts of size 15 cm × 15 cm × 15 cm were prepared by casting. Each concrete mix have different cement replacement ratios. 11 different C25 grade 43 concrete mixtures were cast, of which one was 100% OPC as a controlled group. While preparing the concrete mixture and casting, the slump test was also performed in order to check the workability of the samples. After soaking the concrete in a water bath, a compressive strength test was performed on the 7th and 28th days.

### Characterization technique

The crystalline size and phases of BA, CDP, and binary mixture was recorded by X-ray diffraction (Drawell XRD-7000, China) with CuKα radiations (λ = 1.54 Å) at a scanning speed 0.02° s^− 1^ in 2θ range of 10 to 80° under 30 kV and 25 mA. The functional groups were determined by Fourier transform infrared spectrometer (FTIR, PerkinElmer, spectrum two, USA) in KBr pellet method in the 4000-400 cm^− 1^ with a resolution of 4 cm^− 1^. The chemical composition was examined using X-ray Fluorescence (XRF). The size distribution and polydispersity index (PDI) were measured by dynamic light scattering (DLS) analysis using Nano S (Malvern Instruments, UK).

## Results and discussions

### DLS analysis

**Fig. 4:**
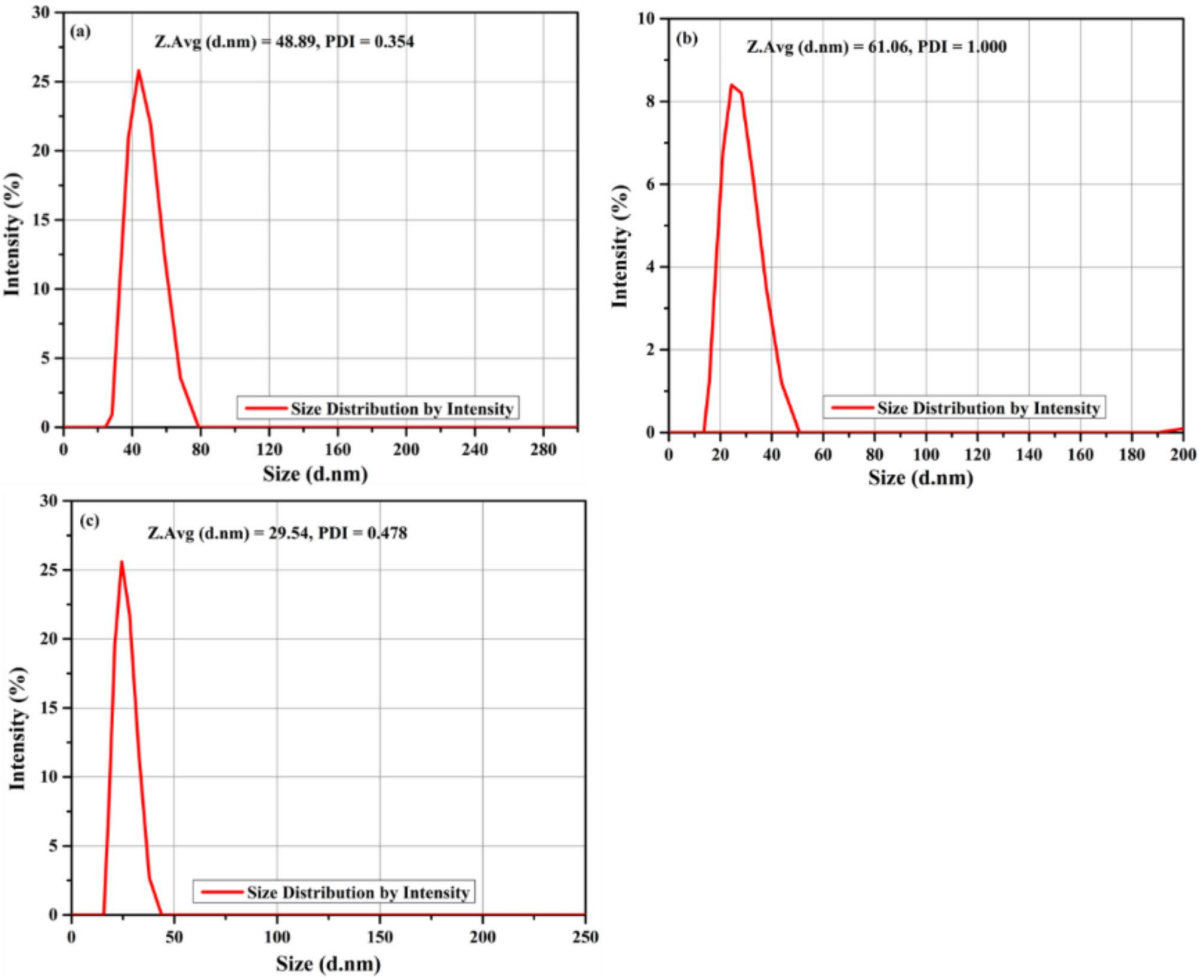
DLS analysis of (**a**) bagasse ash, (**b**) calcined dolomite powder and (**c**) mixture of BA and CDP.

Particle size distribution (PSD) is a crucial physical property influencing the reactivity of pozzolanic materials in concrete mixes, with larger and finer particles generally increasing mix reactivity, thereby optimizing concrete pore structure. Hence, the filling effect of pozzolanic particles has reduced the void between cement and aggregate particles, resulting in improved density and strength ^38^. The PSD of BA, CDP, and a binary mixture were depicted in Fig. 4a-c. The average PSD of CDP was 61.06 nm after fine grinding (Fig. 4a), while the average particle size of BA (Fig. 4b) and their binary mixture (Fig. 4c) was 48.89 nm and 29.54 nm, respectively. Therefore, the result revealed that the pozzolanic activity of grined powders were directly linked to its fineness, with BA and CDP exhibiting good pozzolanic activity ^39–42^.

### XRD analysis

**Fig. 5:**
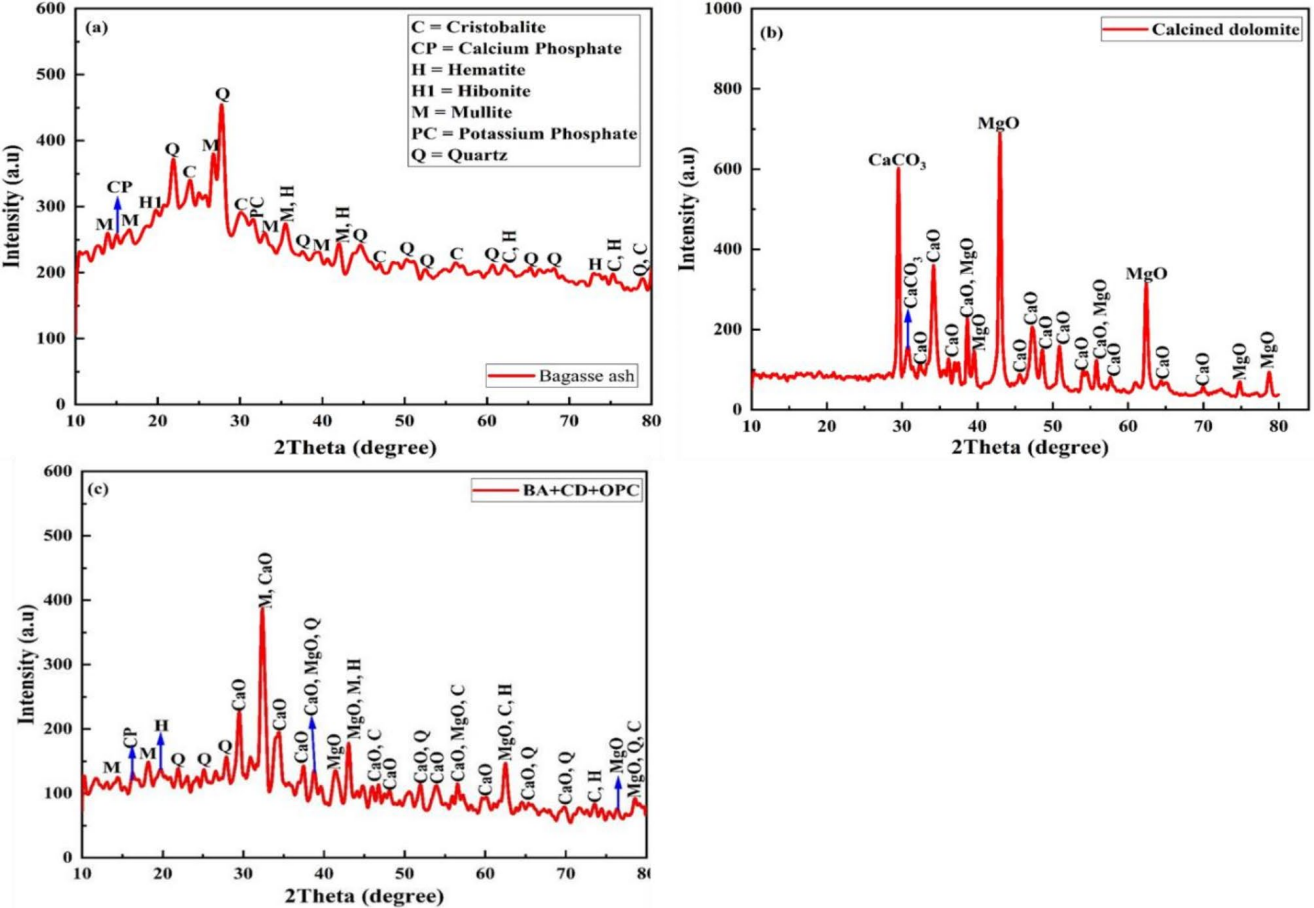
XRD analysis of (**a**) bagasse ash, (**b**) calcined dolomite, (**c**) mixture of BA,CDP andOPC.

**Fig. 6:**
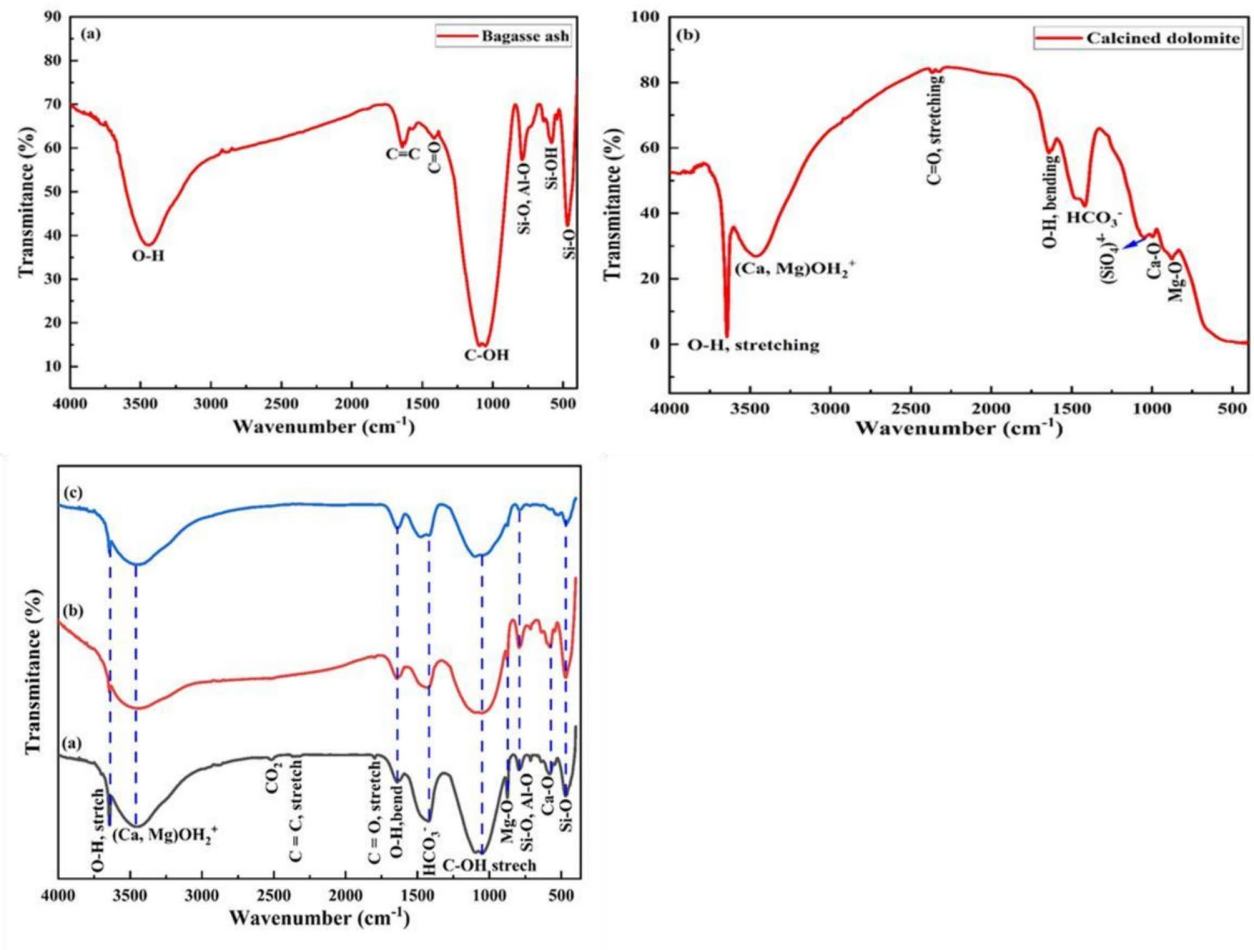
FTIR analysis of (**a**) bagasse ash (**b**) calcined dolomite powder (**c**) mixture of BA, CDP and OPC.

XRD was used to analyze the crystallographic properties of BA and CDP, as shown in Fig. 5a-c. The diffraction pattern of BA was depicted in Fig. 5a. The diffraction peaks of BA were identified as quartz and cristobalite at 2θ values of 20.21 (4.44Å), 22.30 (3.98 Å), 28.10 (3.16Å), 44.98 (2.03Å), 51.27 (1.78Å), 66.81(1.39Å), 25.28 (3.49Å), 30.40 (2.91Å), and 57.15 (1.61Å), respectively. BA has a high concentration of quartz and cristobalite silica, as indicated by the intensity of phase diffraction peaks, which were proportional to the concentration of the component producing it. The oxide composition of BA consists of a cumulative sum of silicon, alumina, and iron oxides, respectively, meeting the chemical requirement of pozzolan as per ASTM standard C618 ^43–46^. On the other hand, XRD pattern of CDP (Fig. 5b) shows sharp picks primarily linked to the presence of CaO and MgO ^47,48^. The XRD pattern of a binary blended mixture revealed the chemical compositions of both BA and CDP, which indicate successfully nixed as a binary system and demonstrated in Fig. 6c.

### Fourier transform infrared analysis (FTIR)

Fig. 6a-c shows the FTIR spectra of BA, CDP, and their ternary mixtures. Fig. 6a depicts the FTIR spectrum of BA. The broad peaks at 3447 cm^−1^ and a strong absorption band between 500 and 1400 cm^−1^ correspond to OH and Si-O symmetric stretching vibration. The SiO₄ symmetric stretching vibration was observed at 820 cm^−1^ and 620 cm^−1^, with the lowest bending mode at 500 cm^−1^. The peak at 1100 cm^−1^ revealed the tetrahedral asymmetric stretching vibration of quartz crystal, as reported by previous work ^49–52^. The absorption peaks observed in CDP were displayed in Fig. 6b. The peak at around 550 cm^−1^ was due to MgO stretching vibration, while the absorption bands observed at 720 cm^−1^, 870 cm^−1^, and 1420 cm^−1^ were due to CaO stretching bonds, and 820 cm^−1^ peaking due to silica impurity. Fig. 6c depicts the presence of various functional groups in the mixture of BA, CDP, and OPC. Water bond (O-H stretching and bending) with hydrated gels of the BA/CDP-OPC reaction was observed at a broad absorption band of around 3460 cm^−1^ and a weak band of 1640 cm^−1^. The O-H broad absorption band intensity of the mixture was found to be lower and broader than BA and CDP due to increased gel formation.

### SEM analysis

**Fig. 7:**
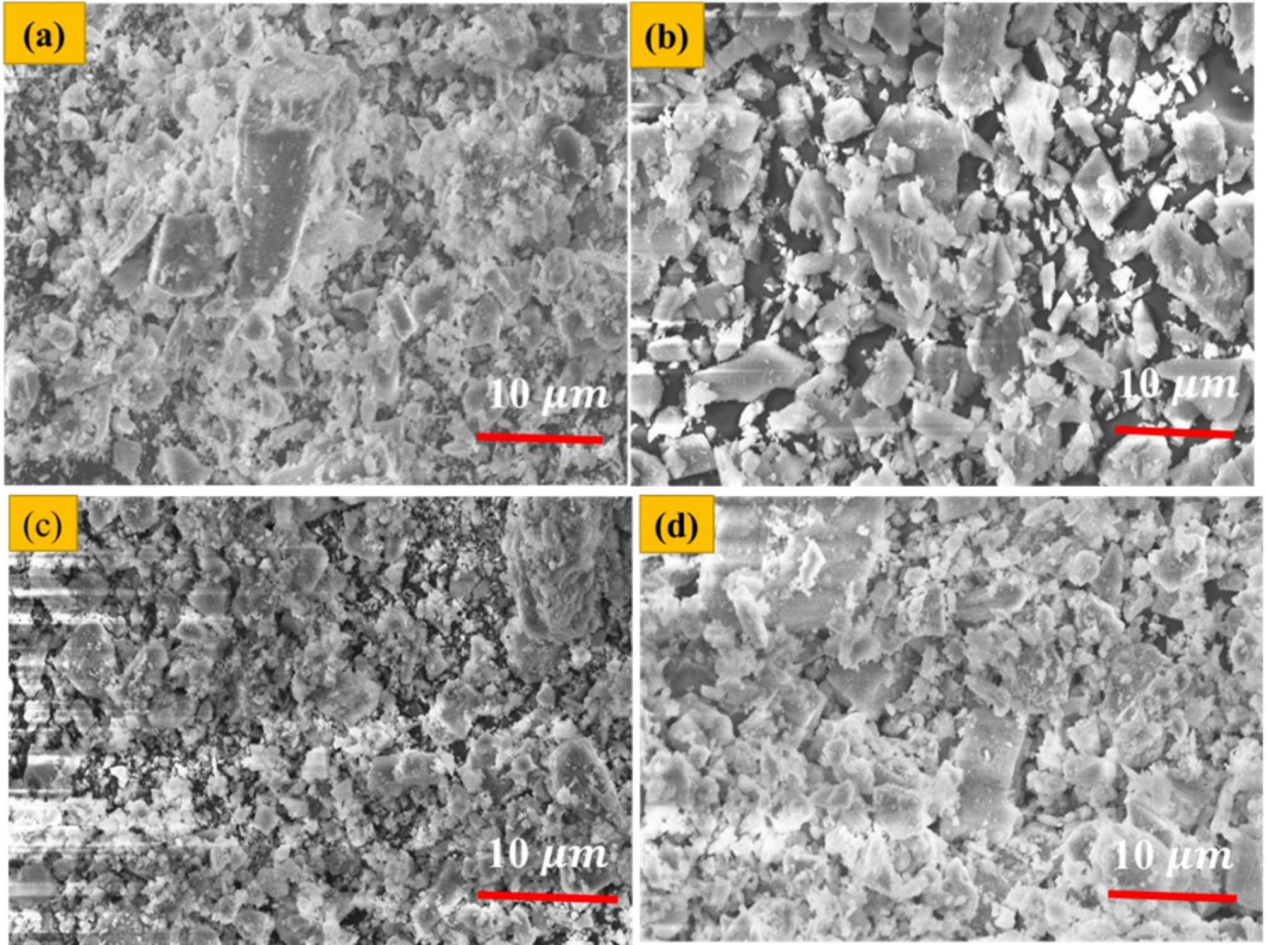
Secondary electron SEM image of (**a**) bagasse’s ash, (**b**) calcined dolomite powder, (**c**) binary mixture of BA and CDP (**d**) mixture of BA, CDP and OPC.

Fig. 7a-d displays SEM images of BA, CDP, binary mixture of BA and CDP, and tertiary mixture of BA, CDP, and OPC. SEM micrographs of BA showed agglomerated, tubular, prismatic, spherical, irregular, porous, and fibrous particles with various shapes (Fig. 7a). The study reveals that the regular shaped particles of BA were silica-rich and have similar morphological features to those reported in the literature regarding their pozzolanic activity ^53^. The silica and alumina present in the BA will react with calcium hydroxide from cement hydration in order to produce additional set of calcium silicate hydrate (Ca-Si-H) or aluminum silicate hydrate which strengthen the concrete ^54^. The efficiency of this reaction depends on the shape and fines of this silica and alumina particles. The SEM image of CDP shows non-uniformly distributed granular structures, as depicted in Fig. 7b. The granular and angular shape of CDP will also contribute for mechanical interlocking within the concrete matrix. CDP is reach in CaO, which will react with silica and alumina in the presence of water to produce Ca-Si-H products. Those the shape and size of CDP also influences the rate this pozzolanic reaction. The binary mixture of BA and CDP (Fig. 7c) display irregularly shaped particles uniformly dispersed, with a small amount of space or void compared to pure CDP, as shown in the SEM image. Fig. 7d, demonstrating the SEM image of the mixture containing BA,CD and OPC, shows the particles are arranged more compactly, leaving fewer voids or empty space between them. This densely packed mixture of BA, CD, and OPC with numerous direct contact points with neighboring particles significantly contributes to the final strength of the concrete.

### XRF analysis

Table 4 displays the chemical compositions of BA, CDP, and the binary mixture. As depicted in Table 4, BA with a significant chemical composition of 42.22% SiO_2_, 15.65% Al_2_O_3_, and 16.90% Fe_2_O_3_ constitutes over 74.77% of the reactive compounds, crucial for its effective pozzolanic activity. Pozzolan’s lower CaO content (1.97%) and OPC’s higher calcium content (62.90%) effectively facilitate successful secondary Ca-Si-H formation during pozzolanic reactions. Furthermore, BA was found to have high alkali content, such as K_2_O (14.62%), thus implying high potential for alkali silica reaction when used in concrete with silica reach aggregates. More importantly, the XRF analysis result of row BA concedes with ASTEM C-618-03 which puts standard specifications for coal fly ash and raw or calcined natural pozzolans for use in concrete. On the other hand, CaO (48.25%) of CDP was greater than BA (1.97%) and binary system (12.60%). This revealed that CDP has the required chemical requirement of raw materials for cement production.

**Table 4:** Chemical composition of BA, CDP, blended mixture and grade 43 OPC.

S. no.	Composition	BA	CD	75%BA/25%CDP	OPC
1	SiO_2_	42.22	3.47	38.54	21.86
2	Al_2_O_3_	15.65	2.79	15.70	5.76
3	K_2_O	14.62	0.10	10.98	0.34
4	Fe_2_O_3_	16.90	0.95	11.30	4.14
5	MgO	1.43	44.17	10.74	1.19
6	CaO	1.97	48.25	12.60	62.90
7	TiO_2_	0.63	0.09	0.49	0.21
8	MnO	1.29	0.10	0.54	0.03
9	Cr_2_O_3_	0.76	–	1.90	0.02
10	P_2_O_5_	3.57	–	0.22	0.41
11	SO_2_	0.31	–	0.11	–
12	SO_3_	–	–	–	2.93

### Workability

**Fig. 8:**
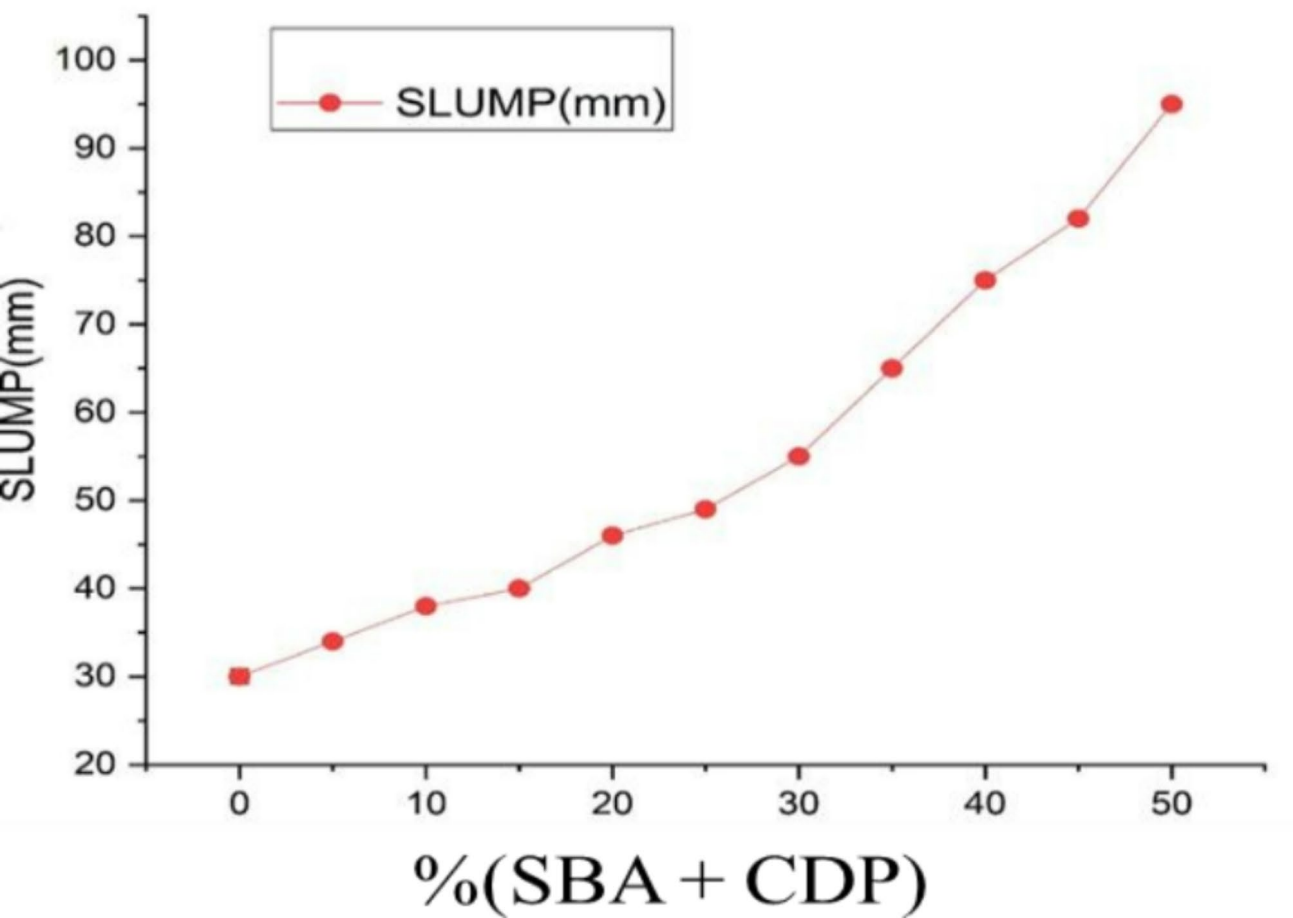
Slump value as the percent of BA and CDP increase.

The slump value of the concrete mixture with a partial replacement of cement was performed according to ASTM 143 to evaluate the workability of the concrete mix ^55^. The value indicated the workability of the concrete mix gradually increased as the percent replacement of cement was increased. The fineness of the BA and CDP particles causes them to occupy a space inside the cement, which reduces the quantity of water needed for a given mixing. Consequently, there was a high level of workability and relative free water, which aligns with previous findings ^56–58^. Fig. 8 shows the slump value of the different percent replacement of cement.

The highest slump obtained was 95 mm, which was for 50% replacement. The lowest slump recorded was 30 mm, which belongs to the 100% OPC. The slump value of concrete mixture increases with the addition of ultrafine BA and CDP due to the small PSD of the binary mixture. The nanoparticles of BA and CDP, due to their small PSD, have a higher surface area than their cement counterparts, thereby improving particle packing and reducing water demand in the mixture. The low water demand leads to a higher slump value for a given mixing.

### Setting time

**Fig. 9:**
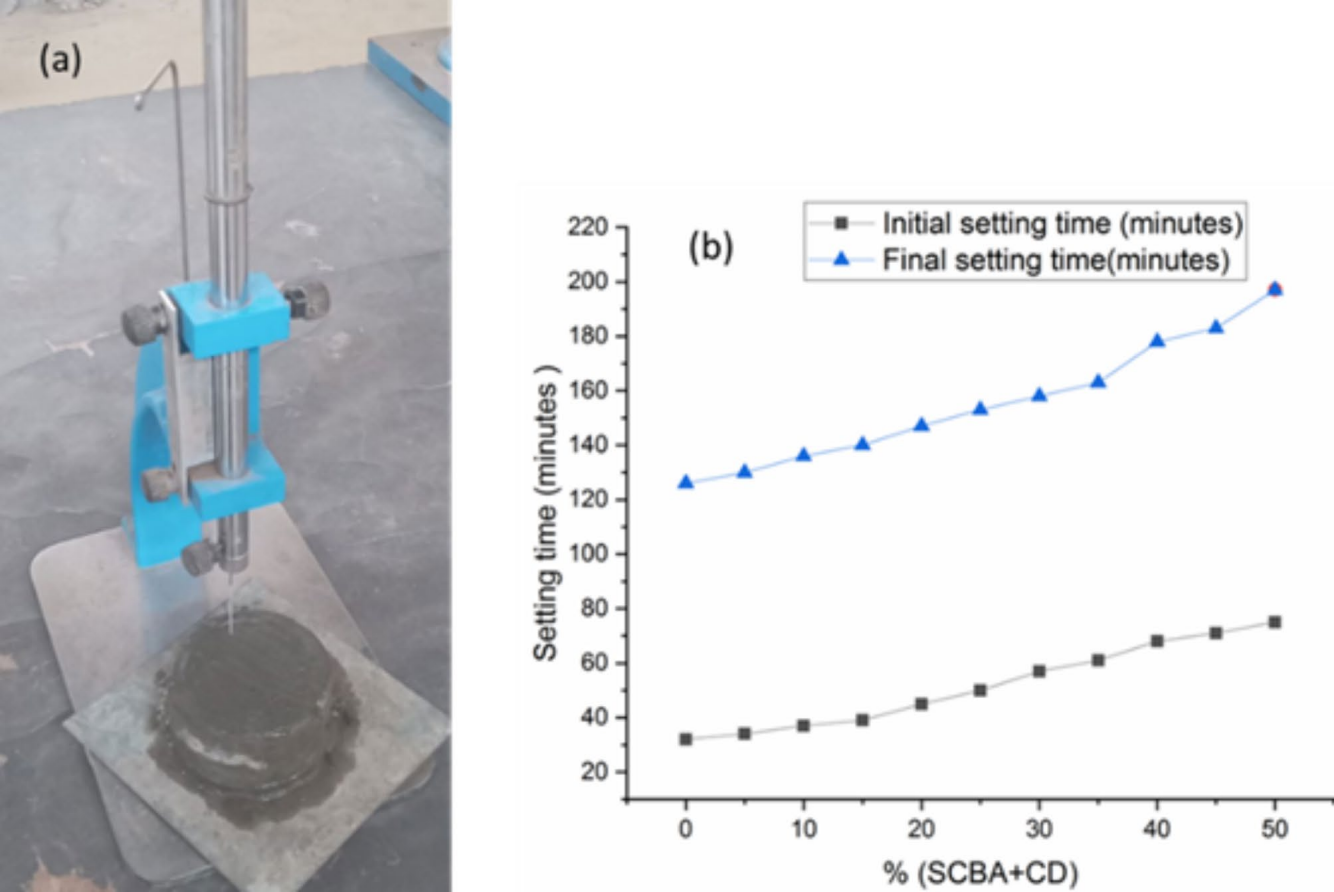
(**a**) The Vicat needle test method (**b**) initial and final setting times for the mix.

The Vicat needle test method, as per ASTM C191, was used to determine the initial and final setting time of cement with varying percent replacements, ranging from 0 to 50%, as depicted in Figs. 9a and b. The result reveals that mixtures containing BA and CDP have higher initial and final setting times compared to a controlled mixture with 100% OPC. This was due to the decrease in OPC available for immediate hydration as the percent replacement increases, as OPC hydrates quickly, leading to shorter setting times (Fig. 9b). This indicates that the initial and final setting times increase with cement replacement ^59,60^. BA, a pozzolanic material, reacts with calcium hydroxide from cement hydration, causing a slower setting process than OPC hydration. CDP, while improving workability and strength, does not set as quickly as OPC, contributing to longer setting times. This trend was similar to previous research on partial cement replacement with BA, indicating a longer setting time ^61–63^.

### Compressive strength

**Fig. 10:**
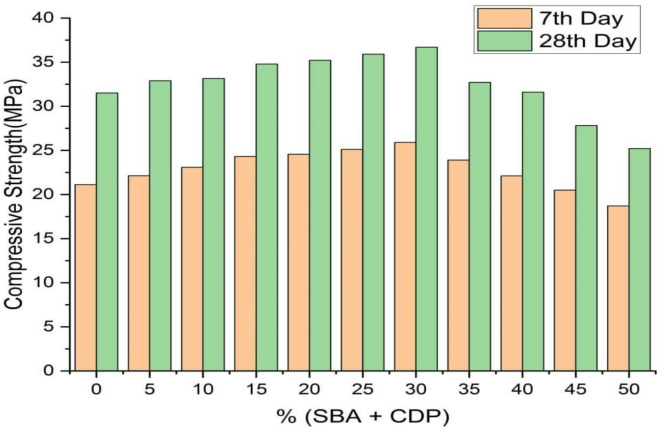
The 7th and 28th days compressive strength value for a partial replacement of OPC with 5% increment of (BA + CDP).

The results of compressive strength tests were shown in Fig. 10. The 30% partial replacement of cement achieved the highest compressive strength, reaching 25.9 at the 7th day and 36.5 at the 28th day from ten different partial replacements. The value of the compressive strength increases as the percent replacement of the cement by the binary mixtures increases until 30% percent. The compressive strength value decreases after 30% due to the increased use of agro-industrial wastes in the mixture of ultrafine CD and BA. Table 5 demonstrates consistent reliability in compressive strength measurements, with low variability and small standard deviation, indicating material stability across cubic samples.

**Table 5:** The 7th and 28th days compressive strength value for a partial replacement of OPC with 5% increment of (SBA + CDP) together with the resulting standard deviation and coefficient of variation.

Cement replacement	Weight of concrete (kg)	7th day Compressive strength value (MPa)	SD (MPa)	COV (%)	28th days Compressive strength value (MPa)	SD (MPa)	COV (%)
100% OPC	8.955	21.12	0.176	0.83	31.5	0.424	1.35
95%OPC + 5% (SBA + CDP)	8.895	22.11	0.23	1.04	32.9	0.041	0.124
90%OPC + 10% (SBA + CDP)	8.98	23.08	0.241	1.04	33.14	0.206	0.618
85%OPC + 15% (SBA + CDP)	8.368	24.31	0.49	2.01	34.79	0.071	0.204
80%OPC + 20% (SBA + CDP)	8.77	24.56	0.5	2.05	35.2	0.46	1.31
75%OPC + 25% (SBA + CDP)	8.674	25.11	0.134	0.53	35.9	0.0943	0.263
70%OPC + 30% (SBA + CDP)	8.799	25.9	0.093	0.36	36.7	0.318	0.866
65%OPC + 35% (SBA + CDP)	8.667	23.9	0.47	1.97	32.7	0.216	0.66
60%OPC + 40% (SBA + CDP)	8.777	22.1	0.212	0.96	31.6	0.285	0.9
55%OPC + 45% (SBA + CDP)	8.432	20.5	0.416	2.03	27.82	1.47	5.29
50%OPC + 50% (SBA + CDP)	8.754	18.7	0.532	2.84	25.2	0.78	3.04

The result also demonstrates the impact of blending BA and CDP on the compressive strength of C-25 grade concrete. The binary mixture results in a higher compressive value than other similar studies using only BA as a cement replacement material, in which the maximum amount of replacement was found up to 15% ^64–66^. The improved compressive strength of a binary mixture of CDP and BA was primarily due to the reduced particle size distribution and appropriate calcination. The compressive strength data shows a 35% replacement of cement, nearly equivalent to 100% OPC with 31 MPa. However, the optimal cement replacement depends on factors like workability, durability, and other performance. A binary system of BA and CDP can replace cement to 30% without affecting concrete performance.

### Strength activity index (SAI)

**Fig. 11:**
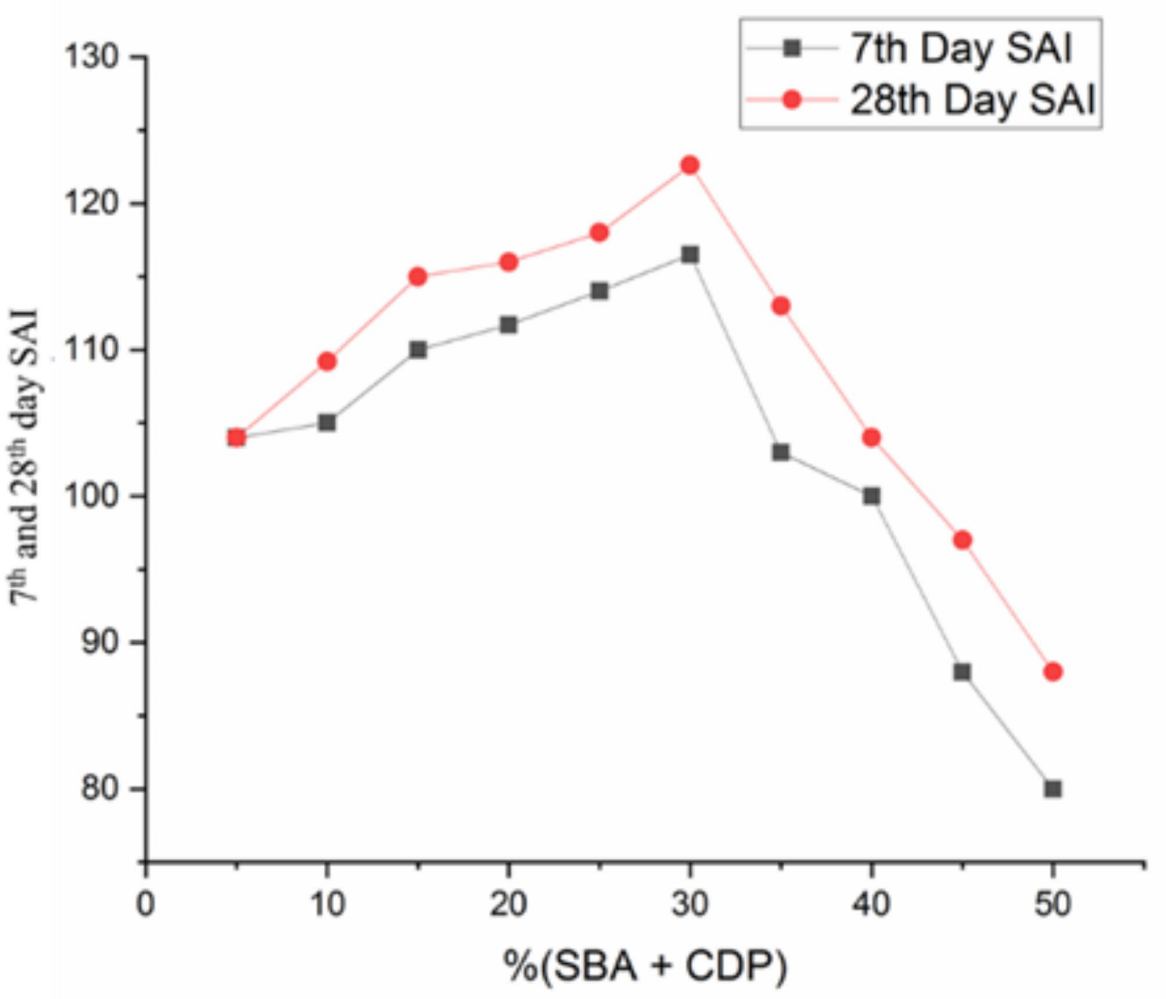
7th and 28th days strength activity index.

The strength activity index (SAI) was determined using compressive strength results from the 7th and 28th days, following ASTM C311, to assess the pozzolanic activity of the binary binder ^67,68^. Fig. 11 shows the strength activity index for the 7th and 28th days, with 30% replacement achieving the highest at 116 and 122, while 50% replacement had the lowest at 80. The activity index indicates that a binary mixture of bagasse ash and calcined dolomite powder is an effective pozzolanic material that surpasses the control mix. The study also reveals that BA blended with CD exhibits high pozzolanic activity, meeting ASTM C311 standards, with a strength activity index above 75 indicating good pozzolanic activity ^69^.

### Water absorption

The Belgian standard NBN B15-215 (1989) measures a material’s water absorption capacity when submerged. This test involves drying and saturating the material with water, assessing concrete’s durability and water resistance, making it a common technique in testing materials as shown in Table 6. The water absorption test revealed that the lowest value was 3.94 for 5% replacement, while the highest was 7.68 for 50% replacement. Concrete mixtures should have a water absorption value between 4 and 6% for longer durability ^70,71^. As cement replacement percentage increases, the water absorption value of the mixture also increases, possibly due to the development of pores due to rising ash content. This increase in ash content also increases the level of specific oxides, such as magnesium and potassium oxide, which are controlled in cement formulations. Magnesium oxide was hydrated to produce magnesium hydroxide, a less dense and higher volume form. This increases the volume of magnesium oxide, causing pours, voids, and micro cracks in concrete structures, resulting in increased porosity and water absorption ^72^. Additionally, excess potassium oxide can cause an alkali-silica reaction between potassium oxide and reactive silica in certain aggregates, leading to disruption, increased porosity, and water absorption due to the expansion of the compound ^73^. This study found that at 30% replacement, maximum compressive strength is achieved due to a balance between packing density and concrete porosity. The optimal packing density and additional C-S-H gel from the pozzolanic reaction resulted in a denser concrete microstructure. However, the increased volume of binary replacement material may have increased overall porosity, affecting water absorption slightly.

**Table 6:** Water absorption value of a concrete with partial replacement of cement with binary system of Bagasse’s Ash and calcined dolomite powder.

Sample name	Initial wait of the dry concrete (g)	Final weight after soaking for 24 h (g)	Water absorption value (%)
95%OPC + 5% (BA + CDP)	2301	2431.5	3.94
90%OPC + 10% (BA + CDP)	2435.5	2531.5	4.128
85%OPC + 15% (BA + CDP)	2337.5	2439.41	4.36
80%OPC + 20% (BA + CDP)	2366.5	2477.48	4.69
75%OPC + 25% (BA + CDP)	2367	2497.89	4.95
70%OPC + 30% (BA + CDP)	2360.6	2484.29	5.24
65%OPC + 35% (BA + CDP)	2335.5	2489.0	6.481
60%OPC + 40% (BA + CDP)	2332.5	2485.5	6.53
55%OPC + 45% (BA + CDP)	2260	2447	7.64
50%OPC + 50% (BA + CDP)	2270.5	2445	7.68

### Dry density

The dry density of the concrete mix was determined using a 10 cm x 10 cm x 10 cm cubic metallic mold with a volume of 0.001 m³. The mass of the dry concrete sample was divided by the concrete volume, as shown in Table 7. The dry density values indicate that the concrete mixture with a partial cement replacement is well-balanced and meets the typical density requirement. The controlled mix has a density of 2357 kgm^-^³, while partial replacement mixtures have a density of 2301 kgm^-^³ for 5% and 2275 kgm^-^³ for 50%. All dry density values fall within the normal acceptable range of 2200 kgm^-^³-2600 kgm^-^³^74^. The 30% replacement of BA and CDP in the concrete mixture resulted in a slight increase in dry density, indicating effective material interaction and packing ^75^. The density of concrete slightly decreases as replacement percentages increase beyond optimum, primarily due to a higher percentage of BA, which may increase porosity, reducing overall density and mechanical performance ^76,77^.

**Table 7:** Dry density values of concretes.

Sample name	Wait of the dry sample (kg)	Dry density (kgm^-^^3^)
100% OPC	2.357	2357
95%OPC + 5% (SBA + CDP)	2.301	2301
90%OPC + 10% (SBA + CDP)	2.435	2435
85%OPC + 15% (SBA + CDP)	2.337	2337
80%OPC + 20% (SBA + CDP)	2.366	2366
75%OPC + 25% (SBA + CDP)	2.367	2367
70%OPC + 30% (SBA + CDP)	2.36	2360
65%OPC + 35% (SBA + CDP)	2.335	2335
60%OPC + 40% (SBA + CDP)	2.332	2332
55%OPC + 45% (SBA + CDP)	2.26	2260
50%OPC + 50% (SBA + CDP)	2.275	2275

## Conclusion

This research involved preparing 11 concrete mixtures and analyzing their compressive strength and freshness, resulting in a major conclusion. The binary mixture of BA and CDP shows superior potential as a pozzolanic material for partial cement replacement, showing more promising results than individual effects. The 30% binary mixture of BA and CDP, when partially replaced with cement, demonstrated superior compressive strength and other properties compared to other mixes, including the controlled mix with 100% OPC. The slump value and initial and final setting time of concrete increased with a 5 to 50% replacement of OPC by the binary blend. The concrete’s performance decreases with a 30% replacement, while increasing it beyond this level results in increased water absorption, negatively impacting its performance. The key parameters contributing to improved concrete performance include grinding techniques for ultra-fine particle size distribution of BA and CDP, calcination temperature, and material blend.

## Recommendation

The research yielded promising results, but further research is needed to expand the field based on the progress made. The study focuses on the compressive strength of concrete, and future researchers should investigate the impact of ultrafine BA and CDP on other mechanical properties. The durability of a concrete mixture containing the binary system also needs further study.

## Data Availability

The datasets generated during and/or analyzed during the current study are available from the corresponding authors on reasonable request.
